# COVID‐19 response in a long‐term care facility for people with epilepsy

**DOI:** 10.1002/epi4.12940

**Published:** 2024-04-09

**Authors:** Luisa Delazer, Noah Pressler, Simona Balestrini, Fenglai Xiao, Lisa M. Clayton, Jonny Anders‐Cannon, Rebecca Salvatierra, Ian Henry, Sanjay M. Sisodiya, Josemir W. Sander, Matthias J. Koepp

**Affiliations:** ^1^ Chalfont Centre for Epilepsy Chalfont St Peter, Bucks UK; ^2^ Department of Clinical & Experimental Epilepsy UCL Queen Square Institute of Neurology London UK; ^3^ Department of Neurology, Epilepsy Center Ludwig Maximilians University Munich Germany; ^4^ University of Nottingham, Medical School Nottingham UK; ^5^ Neuroscience Department, Children's Hospital A. Meyer IRCSS University of Florence Florence Italy; ^6^ Department of Neurology, West China Hospital Sichuan University Chengdu China; ^7^ Stichting Epilepsie Instellingen Nederland (SEIN) Heemstede The Netherlands

**Keywords:** asymptomatic rate, fatality ratio, SARS‐CoV‐2, seizures

## Abstract

**Objective:**

To assess asymptomatic rates and severity of SARS‐CoV‐2 infection in people with epilepsy and their healthcare workers in a long‐term care facility which had implemented weekly surveillance testing between April 2020 and June 2022.

**Methods:**

Questionnaires focused on objective and subjective COVID‐19 symptoms for people with epilepsy residing in and their healthcare workers at the Chalfont Centre for Epilepsy in June 2022. Demographic information, comorbidities, and seizure frequency were gathered from medical records. We also collected responses on objective and subjective COVID‐19 symptoms from healthcare workers who participated in a prospective study assessing the reaction to COVID‐19 vaccinations (SAFER).

**Results:**

Fifty‐five out of 89 (62%) residents tested positive at least once on weekly PCR testing for SARS‐CoV‐2 during the period of interest; 20 of those (37%) were asymptomatic. In comparison, of those 63 healthcare workers who tested positive at least once on weekly testing during the same period, only four (6%) were asymptomatic. Of the 159 healthcare workers who also participated in the SAFER study, 41 tested positive at least once, and seven (17%) were completely asymptomatic during infection with SARS‐CoV‐2.

**Significance:**

People with epilepsy living in a long‐term care facility were more likely to present with asymptomatic SARS‐CoV‐2 infections than healthcare workers at the same facility. Despite possible bias in the reporting of subjective symptoms due to management‐by‐proxy, there is no evidence that vulnerable people living in an epilepsy long‐term care facility showed reduced resilience towards infections.

**Plain Language Summary:**

People with epilepsy living in care home facilities had a surprisingly high degree of asymptomatic infections with SARS‐CoV‐2. Very few residents had severe or fatal outcomes. This is in stark contrast to the widely reported bad outcomes for people without epilepsy in other care homes. People with epilepsy reported significantly less symptoms than their healthcare workers. No changes in seizure frequency during or after infection were observed.

**Key points**

- The rate of asymptomatic infections was significantly higher in people with epilepsy than in healthy controls.
- People with epilepsy reported significantly less frequently objective and subjective symptoms than their healthcare workers.
- No changes in seizure frequency during or after infection were observed.

## 1 INTRODUCTION

People with epilepsy are generally assumed to be a vulnerable group and were advised to seek extra protection throughout the 2020 to 2022 coronavirus pandemic (COVID‐19).^1^ The pandemic negatively affected people with epilepsy with only remote access to care^2^ and early reports during the pandemic associated epilepsy with a higher risk for hospitalization^3^ as well as with increased severity and mortality of severe acute respiratory syndrome coronavirus‐2 (SARS‐CoV‐2) infections.^4^, ^5^, ^6^ However, there is little evidence of increased case fatality, prevalence, or symptom severity of SARS‐CoV‐2 infections among people with epilepsy compared to people with no epilepsy.^7^, ^8^ Similarly, there is no evidence that epilepsy severity is affected by SARS‐CoV‐2 infections.^2^, ^8^

Long‐term care facilities are high‐risk settings for poor outcomes from respiratory disease outbreaks, including COVID‐19, due to the greater prevalence of risk factors like age and chronic health conditions.^9^, ^10^ We audited the health and well‐being of people with epilepsy residing at the Chalfont Center for Epilepsy (CCE), a long‐term care facility for adults with severe epilepsy and comorbidities. In this facility, an enhanced surveillance program was implemented for residents and healthcare workers (HCWs) from the start of the pandemic in April 2020,^10^ allowing us to determine the asymptomatic rate of infections among residents and compare against their HCWs who were likely to be infected by the same strain of the virus. We determined whether the infection or associated symptoms impacted the residents' seizure frequency or severity.

## 2 MATERIALS AND METHODS

On 10 June 2022, residents at the CCE who had previously tested positive for SARS‐CoV‐2 were sent a questionnaire enquiring about the most common symptoms at the time of infection, divided into objective (fever/chills [temperature > 37.8°C, or a temperature rise of 1.5°C above long‐term average], cough, running nose, vomiting, diarrhea) or subjective (sore throat, congestion, nausea, fatigue, muscle or body aches, shortness of breath or difficulty breathing, new loss of taste or smell, headache), or other symptoms (open question). Participants were also asked to scale how badly their physical well‐being was affected during the SARS‐CoV‐2 infection on a Likert scale ranging from 1 (no symptoms) to 10 (burdensome symptoms). An HCW caring for the resident during their infection helped those who could not respond to the questionnaire independently. To reduce reporting bias, daily care records and medical notes of the time of infection were reviewed in detail.

Demographics and data on the infection, frailty score, vaccination status, immunosuppression status, ASMs, etiologies, and the presence of respiratory or heart conditions or diabetes mellitus were taken from medical records. Seizure records were checked for seizure frequencies 6 months before and 6 months after the infection, and a monthly seizure frequency (before and after infection) was estimated.

HCWs working at the CCE between 11 and 16 July 2022 were asked whether they worked at CCE between April 2020 and June 2022 and had tested positive for SARS‐CoV‐2 during this observation period. They were then anonymously given the same symptom questionnaire.

As a second healthy control cohort, HCWs at CCE were invited in January 2021 to participate in a prospective study on high‐risk frontline HCWs in an acute National Health Service hospital trust in London, “SARS‐CoV‐2 Acquisition in Frontline Healthcare Workers—Evaluation to inform Response (SAFER),” to assess the safety and efficacy of COVID‐19 vaccines.^11^ SAFER participants had to respond weekly whether they were showing signs of infection or tested positive. A total of 159 HCWs took part in this study, and data on infections and symptoms during the infection were taken from the SAFER database.

### 2.1 COVID policy and testing

Between 16 March and 17 April 2020, residents were tested for SARS‐CoV‐2 infection only when symptomatic. From 17 April 2020 onwards, all residents (*n* = 89) and all HCWs (*n* = ~400) working at CCE had weekly PCR tests, replaced by weekly lateral flow tests (LFTs) for residents and daily LFTs for HCWs in January 2021. Residents had multiple daily temperature checks. For HCWs, the temperature was checked at the start of each shift or when entering the facility.

Residents and HCWs were offered their first vaccination in December/January 2021, the second in March 2021, and the third in November 2021 (tozinameran or AZD1222). Visitors were forbidden from the site until June 2020, and after that one visit per person per week was allowed, so that residents almost only had contact with HCWs during this period. Due to limited activities outside the facility, asymptomatic HCWs were the most probable transmission source.

### 2.2 Statistical analysis

Statistical analyses were performed using SPSS version 28 (IBM SPSS Statistics). Group comparisons were performed via chi‐square tests and Fisher's exact tests for categorical variables. Depending on data distribution, two sample independent *t*‐tests or Mann–Whitney tests were used to compare continuous variables. When more than two groups were compared, Kruskal–Wallis tests were used (non‐parametric distribution), followed by Bonferroni corrected post hoc tests in case of significant results. A repeated measure ANOVA was applied to assess seizure frequency with seizure frequency before/after infection as within‐subject variable and symptomatic infection/asymptomatic infection as between‐subject variable. Depending on data distribution, descriptive data are presented as mean and standard deviation or median and interquartile range (IQ). *p* < 0.05 was considered statistically significant.

### 2.3 Standard protocol approvals, registrations, and patient consents

This retrospective study was registered as a service evaluation and independently approved by the Clinical Audit and Quality Improvement Subcommittee (UCLH NHS Trust). As such, approval by an ethics committee was not required.

## 3 RESULTS

### 3.1 People with epilepsy

On 10 June 2022, 89 people with epilepsy due to different etiologies had lived at the CCE throughout the pandemic (mean age at the time of data collection: 50.5 years, 32 females, with chronic respiratory (*n* = 17) or cardiac (*n* = 5) conditions, pharmacologically‐induced immunosuppression (*n* = 5), and diabetes mellitus (DM type II, *n* = 1)). At the time of data acquisition, residents took an average of 2.2 (±0.8) antiseizure medications (ASM). In detail, 44 residents received either valproate, lamotrigine (*n* = 38), oxcarbazepine (27), levetiracetam (22), phenytoin (15), carbamazepine (13), topiramate (8), pregabalin (8), zonisamide (7), lacosamide (4), perampanel (2) and/or ethosuximide, phenobarbital, eslicarbazepine, primidone, sultiam, gabapentin. The average frailty score was 5.8 (±1.01).

All residents had a history of drug‐refractory epilepsy, although epilepsy was well controlled in some with a wide range of seizure frequency (see below). Residents also had learning disabilities and/or significant cognitive decline, which would not allow them to live independently.

The 89 residents lived in seven units of 1–4 self‐contained flats, each housing 5–12 people. All seven units reported SARS‐CoV‐2 infections. Each resident had a private bedroom, while living room, kitchen, and bathroom were shared within the flats. During the height of the pandemic, communal rooms were closed, and residents had their meals in their own rooms, with very limited group activities. Residents had access to a large garden and other outdoor spaces. Carer‐to‐resident ratio was 1:1 during daytime and 2:5 during nighttime for the smaller self‐contained flats housing 5 residents, whereas for the larger units, the carer‐resident ratio was 1:2 during daytime and 1:4 during nighttime.

### 3.2 Healthcare workers (HCW)

Sixty‐three HCWs (mean age 46.4 years, 41 females) returned the questionnaires. Forty‐one HCWs (mean age 48.7 years, 28 females) who took part in the SAFER trial tested positive at least once. As questionnaires were returned anonymously, and not all HCWs took part in the SAFER trial, these numbers are not exclusive, and it is not possible to determine the overlap between these two cohorts.

Of the 63 HCWs who returned the questionnaire, five reported a chronic respiratory disease, three reported a cardiac conditions, two were pharmacologically induced immunosuppressed and three HCWs had a diagnosis of DM type II. Of those 41 HCWs who took part in the SAFER trial and tested positive, none reported a chronic respiratory disease, five cardiac condition, none were immunosuppressed and two HCWs had a diagnosis of DM type II.

### 3.3 Infection rates of people with epilepsy and HCWs

Of 89 residents, 55 tested positive (62%, mean age 47.6 years, 19 female) during the period. Infected people with epilepsy were significantly younger than those residents who never tested positive (55.1 years; *p* = 0.026), but did not differ in sex distribution, comorbidities, or other epilepsy‐related variables (number of ASM, valproate intake, seizure frequency).

Thirty‐five residents (63%) were asymptomatic during their first SARS‐CoV‐2 infection. Ten residents had two or more infections, six were asymptomatic on both occasions, three were symptomatic, and one had symptoms only with his first infection. We only ascertained symptoms of the first infection.

Of the 63 HCWs who had tested positive at least once and had returned the questionnaire, 16 (25%) HCWs tested positive at least twice, and all of them but one had a symptomatic first infection.

Of 159 HCWs participating in SAFER, 41 (26%, mean age 48.2 years, 28 female) tested positive at least once, and three tested positive twice. Three HCWs who had two or more infections were symptomatic on all occasions. Thirty‐four (83%) HCWs reported symptoms during their confirmed infection. There was no difference in age and prevalence of comorbidities between the people with epilepsy and these 41 HCWs who tested positive but there were more proportionally more female HCWs than people with epilepsy, in line with general distribution within people with epilepsy living at CCE and the workforce.

### 3.4 Rate of asymptomatic infections in residents and HCW

Based on questionnaire responses, 20 out of 55 (37%) people with epilepsy were asymptomatic compared to 4 out of 63 HCWs (6.4%; *p* < 0.001) and 7 out of 41 HCWs who had taken part in SAFER (17%; *p* = 0.04). Symptomatic and asymptomatic residents did not differ in age, sex distribution, frailty score, presence of comorbidities (heart condition, respiratory condition, diabetes mellitus, immunosuppression) or epilepsy‐related variables (number of ASM, valproate intake, seizure frequency).

Further, symptomatic and asymptotic HCW did not differ in age and sex distribution.

### 3.5 Well‐being and symptom severity in residents and HCW

Using the questionnaire, people with epilepsy reported a median well‐being score of 4 (IQR: 1–6) during infection and HCWs a score of 6 (IQR: 4–7) (*p* < 0.001). The average number of symptoms was significantly higher in this HCWs group (5.22 ± 2.5) than in residents (2.7 ± 2.8; *p* < 0.001). HCWs reported significantly more frequent objective (fever/chills: *p* = 0.01; cough: *p* = 0.003) and subjective symptoms (sore throat: *p* = 0.003; fatigue: *p* = 0.008; muscle or body aches: *p* < 0.001; shortness of breath: *p* = 0.032; loss of taste/smell: *p* < 0.001; headache: *p* < 0.001) than the residents (see Table 1).

**TABLE 1:** Frequency of symptoms in people with epilepsy and healthcare workers (HCWs).

	Symptom	People with epilepsy questionnaire (*N* = 55)	HCW questionnaire (*N* = 63)	HCWs SAFER (*N* = 41)	*p*‐Value (people with epilepsy vs. HCW questionnaire)	*p*‐Value (people with epilepsy vs. HCW SAFER)
					*< 0.05, **< 0.01, ***< 0.001	*< 0.05, **< 0.01, ***< 0.001
	Asymptomatic	20 (36.4%)	4 (6.4%)	7 (17.07%)	***	*
Subjective	Fever or chills	18 (32.7%)	36 (57.1%)	23 (56.09%)	**	n.s.
	Cough	20 (36.4%)	41 (65.1%)	21 (51.21%)	**	n.s.
	Congestion or runny nose	26 (47.3%)	29 (46.0%)	22 (53.65%)	n.s.	n.s.
	Nausea or vomiting	1 (1.8%)	5 (7.9%)	NA	n.s.	NA
	Diarrhea	3 (5.5%)	5 (7.9%)	NA	n.s.	NA
Objective	Sore throat	18 (32.7%)	38 (60.3%)	20 (48.78%)	**	*
	Fatigue	26 (27.3%)	45 (71.4%)	2 (4.87%)	**	***
	Muscle or body aches	14 (25.3%)	43 (68.3%)	27 (65.85%)	***	***
	Shortness of breath	8 (14.6%)	20 (31.8%)	4 (9.75%)	*	n.s.
	New loss of taste or smell	1 (1.8%)	19 (30.2%)	17 (41.46%)	***	***
	Headache	15 (27.3%)	37 (58.8%)	28 (68.29%)	***	***

One 68‐year‐old unvaccinated male resident died, while SARS‐CoV‐2 positive. He had severe drug‐resistant epilepsy and several comorbidities, including dysphagia. Three other residents (55 years, female; 43 years, male, and 57 years, male; all vaccinated) were hospitalized due to worsening of their comorbidities (respiratory condition, endometrial cancer, intestinal condition) while positive. None of them had to be intubated or admitted to intensive care.

Comparing questionnaire responses from people with epilepsy with responses from HCWs taking part in the SAFER trial, HCWs reported more frequently objective (fever/chills: *p* = 0.014) and subjective symptoms (sore throat: *p* = 0.036; fatigue: *p* < 0.001; muscle or body aches: *p* < 0.001; loss of taste/smell: *p* < 0.001; headache: *p* < 0.001) (see Table 1).

Comparing the two HCWs groups, HCWs responding to the questionnaire reported more often subjective symptoms than HCWs taking part in SAFER (fatigue (*p* < 0.001), muscle or body aches (*p* = 0.003), shortness of breath (*p* = 0.01)). All other symptom frequencies did not differ between the two HCW groups.

### 3.6 Association of vaccinations and infection rates

All 89 residents were vaccinated at least once during the pandemic. At the time of testing positive, 17 were unvaccinated, five had one vaccination, and 33 had three inoculations. The asymptomatic rates were not affected by vaccination status.

We did not obtain information about vaccination status using the questionnaire, but all participants of the SAFER trial were vaccinated at similar time points as residents.

### 3.7 Seizure frequency in residents

There was no change in monthly seizure frequency associated with the infection (average monthly seizure frequency before: 10.7 ± 12.8, after: 10.8 ± 12.6) or the presence of symptoms during the infection. The two factors (before/after infection and the presence of symptoms) did not interact. Only one person (1.8%) had a seizure exacerbation during their infection (compared to the seizure frequency 6 months before) and was precautionarily admitted to a ward. The rate of asymptomatic infections did not differ between eight residents who were seizure‐free 6 months before the infection and residents who had at least one seizure in this period. Of note, no increase in seizure frequency was noted during and after the infection in three people with Dravet syndrome.

## 4 DISCUSSION

We systematically assessed infection rate, symptom severity, and seizure frequency related to SARS‐CoV‐2 in people living in a long‐term care epilepsy facility. Through close surveillance and weekly testing, we determined an asymptomatic rate of 37%, which was surprisingly high among residents with epilepsy. The proportion of asymptomatic infections found in our resident sample is comparable to that reported for healthy people in a large meta‐analysis.^12^ In comparison, the asymptomatic infection rate among HCWs was lower, and the number of objective and subjective symptoms was significantly higher than for residents.

In line with the number of symptoms reported during infection, residents were less compromised in their well‐being than their care workers. Neither the infection nor the infection‐related symptoms impacted monthly seizure frequency. Vaccination did not affect the frequency of reported symptoms in residents.

Our results are in stark contrast to the high mortality in other long‐term care facilities for individuals suffering from comparable fragility and co‐morbidities.^9^, ^13^ Whilst it is likely that this is in part explained by the higher mean age of residents in nursing and residential homes compared to that in the CCE, there was no difference in age between people with epilepsy (47.6 years) and HCW (48.2 years) testing positive at the CCE.

Weekly surveillance testing and early identification of infection with immediate isolation were likely the main factors, differentiating CCE from other long‐term care facilities with much poorer outcomes. This likely resulted in lower transmission rates and reduced viral load for residents. Due to close surveillance, we could identify asymptomatically infected HCWs early. Thus, we were able to prevent the asymptomatic from further spreading the infection, considered the “Achilles' heel” of the pandemic.^14^ Close surveillance was likely essential for the containment of the infection rate.^10^ Thus, we suggest that instead of weekly testing, daily testing in those residents who tolerate this procedure may lead to even better outcomes in similar future situations.

Our results suggest a favorable outcome for residents during the pandemic. The overall positive outcome for vulnerable people with epilepsy living in a high‐risk environment is surprising. Similarly, low‐case fatality rates and ICU admission rates were reported for people with epilepsy comparable to healthy people.^7^ In contrast, a Korean study reported higher risks of severe complications in people with epilepsy than in people without epilepsy.^6^

Of interest is the outcome for people with Dravet syndrome who may have temperature‐sensitive seizures. As noted in our earlier small‐scale report and observed in our study, the response to vaccination and the response to infection was not different between the residents with Dravet syndrome and those due to other aetiologies.^15^

We hypothesize that people with epilepsy may be better prepared for infections than others without epilepsy. Chronic epilepsy is associated with chronic inflammation, C‐reactive protein (CRP) levels are raised,^16^ and seizure‐induced activation of the innate immune system^17^ and inflammatory molecules likely provide a tissue environment unfavorable for viral infection and replication.

This may give the residents greater resilience against the SARS‐CoV‐2 virus.^18^, ^19^ Such modifications have been reported in peripheral immune cells and brain tissue from people with epilepsy and have also been reported in large‐scale gene expression profiling.^20^ Consequently, epilepsy may lead to greater resilience as regards the confrontation with the SARS‐CoV‐2 virus in affected individuals.

Another explanation for the better outcome of residents compared to their carers is the limited exposure, in particular during the initial lockdowns. Communal areas were closed and residents had their meals in their own rooms. Rules were more relaxed once all residents and most of the carers had been vaccinated.

A significant limitation of this audit is the retrospective approach and subjective nature. The classification of most symptoms depends on the subjective impression and memory of the infected individual. Similarly, the rating of severity of symptoms and well‐being is necessarily subjective. Many residents are managed by proxy and cannot describe subjective symptoms, and reporting symptoms relies on the impression of the responsible care worker and daily medical records. Of note, objectively measurable symptoms, such as fever and cough were also more frequent among infected HCWs than residents.

There could also be a referral bias, though, as we could not contact all HCWs who tested positive. They were asked to return questionnaires focused on symptoms of COVID‐19 likely to lead to an underestimation of the asymptomatic infection rate, as shown in the difference between questionnaire responses and in prospective SAFER trial. A further limitation presents the frequent change of HCWs during the pandemic. Thus, we were not able to determine the exact number of SARS‐CoV‐2‐positive cases in about 400 HCW during the time of interest. Of note, the sex distribution was not balanced between the people with epilepsy group and the HCW cohorts. Since we did not observe any differences in the asymptomatic rate between men and women within the three cohorts, a sex bias seems unlikely.

Finally, we did not have information of virus variant classifications, which could allow a better analysis of viral effects on people with epilepsy. Since they lived in isolation (no or very few outside visits were permitted), there is a high probability that infections were transmitted from HCW to residents. We thus assume that people with epilepsy and HCW were affected by the same virus variants at specific periods.

## 5 CONCLUSION

In conclusion, we observed an unexpectedly high degree of asymptomatic infections with SARS‐Cov‐2 in residents with epilepsy living in a long‐term care facility. Neither the infection nor the related symptoms were associated with a change in seizure frequency. We hypothesize that chronic inflammation and activation of innate immune responses triggered by recurrent seizures might neutralize SARS‐CoV‐2 more rapidly, eliminating the virus faster and resulting in either asymptomatic or mild infections.

## AUTHOR CONTRIBUTIONS

LD collected the data, conducted statistical analyses, drafted the manuscript, and designed Table 1, NP conducted statistical analyses and drafted the manuscript, SB collected data and critically revised the manuscript, FX collected data and critically revised the manuscript, LC collected data and critically revised the manuscript, JAC helped in the data collection and critically revised the manuscript, IH helped in the data collection and critically revised the manuscript, SMS critically revised the manuscript, JWS critically revised the manuscript, MJK is responsible for the study design and critically revised the manuscript.

## CONFLICT OF INTEREST STATEMENT

The authors do not have any financial interest to disclose.

## FUNDING INFORMATION

This audit was conducted at the University College London Hospital Comprehensive Biomedical Research Centre, which receives a proportion of funding from the UK Department of Health's National Institute for Health Research Centres funding scheme. None of the authors report conflicts of interest. JWS receives research support from the Dr Marvin Weil Epilepsy Research Fund, UK Epilepsy Society, and Christelijke Vereniging voor de Verpleging van Lijdersaan Epilepsie, and reports personal fees from Eisai, UCB Pharma, and Angelini Pharma and grants from Eisai, UCB, and Jazz Pharma, outside the submitted work. MJK served on a scientific advisory board of BIAL and Jazz Pharmaceuticals and has received honoraria for lectures from Eisai, Bial, Novartis, and UCB Pharma.

## ETHICS STATEMENT

We confirm that we have read the Journal's position on issues involved in ethical publication and affirm that this report is consistent with those guidelines.

## Data Availability

The data supporting this study's findings are available on reasonable request from the corresponding author. The data contain information that could compromise participants' privacy, so they are not publicly available.
